# Academic Emotions Under Pressure: Developmental Trajectories and the Role of Shift-and-Persist in High School Students

**DOI:** 10.3390/bs16071063

**Published:** 2026-06-26

**Authors:** Bingxin Cai, Hengchang Huang, Xuhai Chen

**Affiliations:** 1 School of Psychology, Shaanxi Normal University, Xi’an 710062, China; bingxin@xynu.edu.cn (B.C.); huanghc@snnu.edu.cn (H.H.); 2 Mental Health Education Center, Xinyang Normal University, Xinyang 464000, China

**Keywords:** high school students, academic emotions, shift-and-persist, latent growth modeling, cross-lagged panel modeling

## Abstract

High school is often an academically demanding environment that exposes adolescents to elevated emotional challenges. Yet the development of academic emotions across the full high school period, as well as the potential protective role of adaptive coping strategies such as shift-and-persist, remains insufficiently understood. To address this gap, the present study tracked 608 students (305 males; *M*_age_ = 15.56, *SD* = 0.58) from a public senior high school in Chongqing, China, over three consecutive years. Latent growth modeling showed that negative academic emotions increased steadily, whereas positive emotions remained relatively stable. Cross-lagged panel analyses further revealed that greater use of shift-and-persist strategies—a coping approach integrating cognitive reappraisal with optimistic persistence—was prospectively associated with more favorable emotional outcomes, reflected in higher positive and lower negative emotions. In contrast, academic emotions did not predict later use of S-P. These findings point to the cumulative emotional challenges adolescents face in competitive academic settings and highlight S-P as a stable psychological resource. The study extends the control-value framework by identifying a cognitive-affective pathway associated with emotional well-being and emphasizes the potential value of promoting S-P-related coping skills within educational settings.

## 1. Introduction

Academic emotions refer to the emotions that students experience within the school context (Pekrun et al., 2002). These emotions are closely associated with high school students’ cognitive functioning, academic achievement, and psychological well-being (Chang et al., 2020; Hayat et al., 2020). Prior cross-sectional studies suggest that students in higher grades typically report fewer positive and more negative academic emotions than their younger peers (Jin et al., 2022; Raccanello et al., 2013). Longitudinal investigations of school transition periods further reveal characteristic emotional changes, showing that positive emotions tend to decline while negative emotions increase during critical transitions such as the move from elementary to middle school or from middle to high school (Meyer & Schlesier, 2022; Vierhaus et al., 2016; Wang et al., 2015). Despite these findings, the developmental trajectory of academic emotions across the entire high school stage remains insufficiently understood. Existing evidence from both Western and East Asian contexts suggests that academic emotions are closely linked to students’ learning experiences and psychological adjustment. For example, studies conducted in Germany, Finland, and the United States have documented significant associations between academic emotions, academic achievement, and well-being (Pekrun, 2017; Wang et al., 2015). Similarly, research in China has shown that academic emotions are closely related to students’ academic engagement, achievement, and mental health outcomes (Chang et al., 2020; Jin et al., 2022). However, compared with many Western educational systems, Chinese high school students experience particularly sustained academic pressure due to the highly competitive National College Entrance Examination (Gaokao), which spans three years and intensifies as students progress through high school. Consequently, their emotional trajectories may exhibit distinctive developmental patterns and potentially steeper changes than those observed in more general adolescent samples.

When high school students face inescapable academic stress, adaptive coping strategies such as shift-and-persist may be important psychological resources associated with academic emotions (Rood et al., 2012). Shift-and-persist refers to a coping approach in which individuals facing chronic stress adjust by accepting reality and cognitively reappraising adverse situations, while simultaneously sustaining a hopeful outlook and deriving personal meaning to navigate ongoing challenges (E. Chen & Miller, 2012; X. Y. Hu et al., 2022). Previous studies have shown that this strategy mitigates the adverse impact of financial hardship on physical health outcomes such as asthma, obesity, diabetes, and inflammation (E. Chen et al., 2012, 2015; Kallem et al., 2013; Mello et al., 2020). It also alleviates symptoms of depression, anxiety, and PTSD following natural and social disasters (Lopez-Cepero et al., 2024), and fosters positive psychological functioning and the sense of life fulfillment (Benner et al., 2024; Lopez-Cepero et al., 2024; Stein et al., 2022). However, whether shift-and-persist strategies similarly facilitate the positive development of academic emotions in high school students remains understudied.

Despite growing interest in academic emotions and adaptive coping strategies, two important gaps remain in the literature. First, most previous studies have relied on cross-sectional designs or focused on specific school transition periods, leaving the developmental trajectories of academic emotions across the entire high school stage largely unexplored. Second, although shift-and-persist has been linked to a range of psychological and health-related outcomes, little is known about its role in the longitudinal development of academic emotions. Furthermore, the directional longitudinal associations between shift-and-persist and academic emotions have not been systematically examined. Addressing these gaps may contribute to a better understanding of how adolescents adapt emotionally to sustained academic pressure during high school.

To address these gaps, the present study conducted three waves of data collection—one per year, beginning in the first year of high school, with a sample of 608 students, to capture changes in students’ academic emotions throughout high school. Latent growth modeling was used to depict their developmental course, while shift-and-persist strategies and gender were examined as covariates influencing these trajectories. In addition, cross-lagged panel models tested whether the use of shift-and-persist strategies predicted later emotional experiences, and whether academic emotions, in turn, shaped subsequent use of these strategies.

### 1.1. Academic Emotions and Their Developmental Trajectories

Researchers commonly classify academic emotions into two categories: positive and negative (Pekrun et al., 2002). Positive academic emotions, which reflect favorable experiences during academic activities, have been shown to positively predict academic achievement (Pekrun, 2017), facilitate interpersonal relationships and school adjustment (Chang et al., 2020), and enhance metacognitive abilities (Hayat et al., 2020; Fredrickson, 2001). In contrast, negative academic emotions, which reflect unpleasant emotional experiences (Watson, 1988), are significantly associated with depression and academic procrastination (Chan et al., 2015; L. Liu et al., 2023).

The developmental trajectory of academic emotions during adolescence remains a topic of debate. Cross-sectional studies have reported that older students tend to exhibit higher levels of negative academic emotions than younger students (Raccanello et al., 2013). Evidence from a Chinese sample indicates that second-year high school students exhibit higher levels of both positive and negative emotions compared with first-year students (Jin et al., 2022). This trend—of academic emotions becoming more negative with advancing grade level—has also been supported by longitudinal studies. Meyer and Schlesier (2022) and Vierhaus et al. (2016) observed a decline in positive emotions and an increase in negative emotions during the transition from elementary to secondary school. Similarly, Pekrun et al. (2007) reported a significant decline in positive emotions related to mathematics during this period. A comparable pattern was found among Finnish students progressing from lower-level to upper-level secondary education (Wang et al., 2015).

However, some studies suggest that negative academic emotions remain relatively stable in the short term, without showing clear developmental changes over time (Blanke et al., 2022; M. Kim et al., 2024). Notably, most existing findings are based on cross-sectional data or short-term longitudinal designs. Thus far, the developmental course of academic emotions across the full high school period has not been systematically investigated. Within the Chinese educational system, high school students encounter exceptionally heavy academic workloads, along with rigorous evaluative standards. As they advance to higher grades, the pressure they perceive often intensifies. Consequently, their negative academic emotions may increase with grade level, whereas positive academic emotions may decline or remain relatively stable.

### 1.2. Academic Emotions and Their Association with Gender and Shift-and-Persist

Academic emotions develop through the interaction of environmental and individual factors. On the environmental side, influences such as family functioning, peer relationships, teachers’ feedback and a sense of school belonging have been identified as key determinants (Cheng et al., 2025; Wilkinson-Lee et al., 2011). Recent studies have further highlighted the importance of family and school contexts in adolescents’ emotional adjustment (Angelini et al., 2026; Zhu et al., 2023). In parallel, individual characteristics—including academic self-efficacy (Hayat et al., 2020; R. D. Liu et al., 2018), self-control (King & Gaerlan, 2014), emotion regulation strategies (Rood et al., 2012), psychological resilience (Lv et al., 2017), and gender (Gentry et al., 2002; Goetz et al., 2013; Pekrun, 2017)—further shape how students experience and regulate their academic emotions.

Gender differences represent an important focus in the study of academic emotions. Evidence shows that in subjects such as mathematics, female students often report higher levels of anxiety and lower levels of enjoyment than their male peers (Goetz et al., 2013; Pekrun, 2017), although other findings suggest that such differences are not consistent across domains (Gentry et al., 2002). A recent longitudinal study of high school students further demonstrated that girls experienced greater negative emotions than boys when facing examination stress (Huang et al., 2026). These findings suggest that gender effects on academic emotions may vary by context and developmental stage, underscoring the importance of including gender as a covariate in longitudinal research.

However, the most extensively discussed and among the most influential individual factors are intrinsic psychological characteristics. The coping strategy known as “shift-and-persist” may be particularly important for supporting the emotional development of high school students. As a form of positive psychological coping, shift-and-persist is closely associated with the capacity to regulate emotional responses under stress. The “shift” component refers to emotion regulation strategies such as acceptance and cognitive reappraisal. Cognitive reappraisal, which involves positively reframing emotionally challenging situations, is considered one of the most effective emotion regulation strategies (Gross, 1998). It is associated with greater positive affect (Brockman et al., 2017) and reduced vulnerability to negative emotional states (Rood et al., 2012). Other evidence suggests that pairing reappraisal with acceptance reduces the intensity of stress reactions, encompassing both emotional and physiological dimensions (Hayes et al., 2006; Troy et al., 2018; Wolgast et al., 2011). The “persist” component involves enduring adversity with strength, maintaining optimism about the future, and finding meaning in life (E. Chen & Miller, 2012). A strong sense of meaning helps individuals cope with trauma (S. H. Kim et al., 2011) and provides psychological support during physical health challenges (Dezutter et al., 2013; Knizek et al., 2024). Moreover, individuals with a clear sense of life purpose tend to be more resilient to negative emotions (Schaefer et al., 2013).

Empirical evidence suggests that shift-and-persist can buffer the negative emotions of socioeconomic adversity on physical health outcomes, including asthma, obesity, diabetes, and inflammation (E. Chen et al., 2012; Kallem et al., 2013; Lam et al., 2018; Mello et al., 2020), and promote healthier cortisol regulation (L. Chen et al., 2019). Psychologically, this coping strategy reduces symptoms of depression, anxiety, and PTSD following exposure to natural disasters or social stressors (Lopez-Cepero et al., 2024), lowers suicide risk among adolescents with a history of childhood trauma, and mitigates the harmful effects of peer and racial discrimination (Christophe et al., 2019; Santo et al., 2024; Zhang et al., 2023). It also enhances overall psychological well-being and life satisfaction (Benner et al., 2024; Lopez-Cepero et al., 2024; Stein et al., 2022). The development of shift-and-persist strategies begins in childhood, primarily through parental modeling (E. Chen & Miller, 2012; Christophe et al., 2024). By demonstrating appropriate social behaviors, fostering secure attachment relationships, and explicitly teaching coping strategies, parents facilitate the development of adaptive emotion regulation (Lee et al., 2024; Mello et al., 2020).

The control-value theory of academic emotions proposes that adolescents’ academic emotions are influenced by both external contexts, including family, school, and society (Wilkinson-Lee et al., 2011), and internal factors such as perceived control—the belief in one’s ability to accomplish academic tasks—and subjective value, the importance and meaning assigned to those tasks (Pekrun et al., 2007). More specifically, the two components of shift-and-persist may be related to academic emotions through somewhat different psychological processes. The shift component, which involves acceptance and cognitive reappraisal, may help reduce negative academic emotions by decreasing the perceived threat of academic stressors and promoting more adaptive interpretations of challenging situations (Ford et al., 2018; Grol & De Raedt, 2021). In contrast, the persist component, characterized by optimism and meaning-making, may contribute to the maintenance or enhancement of positive academic emotions by strengthening students’ sense of purpose, future orientation, and perceived value of academic activities (Joshanloo, 2024; Yukhymenko-Lescroart & Sharma, 2022). Together, these processes may facilitate emotional adjustment in academically demanding environments. When high school students encounter academic stress or adversity, the use of shift-and-persist strategies—accepting challenging circumstances, cognitively reframing them in a more positive light, maintaining optimism, and seeking meaning—can enhance their sense of control and foster more frequent experiences of positive academic emotions. Sustaining optimism and recognizing the long-term value of learning further contribute to emotional engagement. From this perspective, shift-and-persist may play a crucial role in regulating the dynamic balance of academic emotions during adolescence. Based on the Broaden-and-Build Theory of Positive Emotions, such affective states expand individuals’ thought-action repertoires, promoting adaptive coping through greater cognitive flexibility and the cultivation of personal resources (Fredrickson, 2001). Academic emotions may not only be influenced by using shift-and-persist strategies but may also reinforce their application, forming a mutually reinforcing cycle.

### 1.3. The Present Study

To investigate the developmental trajectories of academic emotions over the three years of high school and to examine the role of gender and shift-and-persist strategies in shaping these trajectories, we conducted a three-wave longitudinal study involving 608 high school students, with data collected annually. We first applied latent growth modeling to assess longitudinal changes in positive and negative academic emotions. We then included gender and shift-and-persist strategies as covariates to evaluate their impact on these emotional trajectories. Finally, we used cross-lagged panel modeling to explore the directional longitudinal associations between academic emotions and shift-and-persist strategies.

Based on the above literature, we proposed the following hypotheses:

**Hypothesis 1.**  *Negative academic emotions will increase across the high school years, whereas positive emotions are expected to remain stable or decline.*

**Hypothesis 2.**  *Gender and shift-and-persist strategies will account for individual differences in the initial levels and developmental patterns of academic emotions.*

**Hypothesis 3.**  *Greater use of shift-and-persist strategies will predict higher subsequent positive emotions and lower subsequent negative emotions. The reverse predictive pathway, from academic emotions to later shift-and-persist strategies, is also explored.*

## 2. Methods

### 2.1. Participants and Procedures

The present study employed cluster sampling to conduct three annual follow-up assessments with first-year students from a public senior high school located in a suburban district of Chongqing, China. Assessments were conducted annually starting in January 2021. The initial assessment (T1) included 1055 participants, followed by 820 participants at the second assessment (T2), and 637 participants at the third wave (T3). After excluding unmatched or invalid questionnaires, 608 participants were retained for longitudinal analyses.

The sample included 305 male and 303 female students, whose average age at T1 was 15.56 years (*SD* = 0.576). Information on parental educational attainment was collected as part of the demographic questionnaire. The majority of parents had completed high school education or below (fathers: 87.0%; mothers: 92.3%), while a smaller proportion held a bachelor’s degree or above (fathers: 13.0%; mothers: 7.7%).

Results from Little’s MCAR test indicated that the missing data were completely at random, *χ*^2^(57) = 57.76, *p* = 0.45. Attrition analyses revealed no significant differences between retained and dropped participants in gender, age, positive academic emotions, negative academic emotions, or shift-and-persist (gender: *χ*^2^(1) = 0.63, *p* = 0.43; age: *t* = −0.44, *p* = 0.66; positive emotions: *t* = 0.32, *p* = 0.74; negative emotions: *t* = 0.35, *p* = 0.73; shift-and-persist: *t* = −0.22, *p* = 0.83).

Participation was voluntary, and respondents were assured that their information would be kept confidential and analyzed only for scientific purposes. The study was approved by the Institutional Ethics Committee of our university (Ethical Review Approval Number: HR 2021-1-050). Prior to data collection, written informed consent was obtained from all participating students, their parents or legal guardians, and the teachers responsible for coordinating the assessments. Students were informed that participation was voluntary and that they could withdraw from the study at any time without penalty. During the testing phase, a trained mental health teacher in each class acted as the main examiner, reading standardized instructions aloud and asking students to complete the questionnaire independently.

### 2.2. Measurement

#### 2.2.1. Shift-and-Persist

Shift-and-persist was measured using the 8-item scale developed by E. Chen and Miller (2012). The scale assesses an individual’s tendency to adapt to chronic stress through positive cognitive and emotional coping. Previous studies have demonstrated satisfactory reliability and validity of the measure among adolescent populations (E. Chen et al., 2015; Murphy et al., 2023). Participants rated each item on a 4-point scale ranging from 1 (never) to 4 (always). A sample item is “I feel that my life has a sense of purpose.” Higher scores indicate greater use of shift-and-persist coping strategies. Based on the T1 data, a confirmatory factor analysis supported the one-factor structure of the Shift-and-Persist Scale, *χ*^2^(13) = 42.17, CFI = 0.974, TLI = 0.958, RMSEA = 0.061, and SRMR = 0.033. Standardized factor loadings ranged from 0.646 to 0.788. McDonald’s omega coefficient was 0.760, indicating acceptable reliability. Together, these results support the structural validity of the scale. Cronbach’s α coefficients were 0.805 (T1), 0.828 (T2), and 0.817 (T3), indicating good internal consistency across all three waves.

#### 2.2.2. High School Students’ School Emotional Experience Scale

Students’ emotional experiences were assessed using a seven-item School Emotional Experience Scale adapted from the Secondary Students’ School Living Experiences Questionnaire developed by the Assessment Research Centre of the Hong Kong Institute of Education (now The Education University of Hong Kong; Assessment Research Centre, 2010). The scale consists of seven items measuring two dimensions of school-related emotional experiences: positive emotional experiences (4 items) and negative emotional experiences (3 items). Positive emotional experiences assess students’ feelings of happiness, accomplishment, security, and being cared for at school, whereas negative emotional experiences assess feelings of boredom, frustration, and the perception that time passes slowly during school activities. Participants rated their agreement with each statement on a 4-point Likert scale ranging from 1 (Strongly Disagree) to 4 (Strongly Agree). Although the scale assesses emotional experiences within the school context, its two-dimensional structure is conceptually consistent with the distinction between positive and negative academic emotions proposed by Pekrun et al. (2002). Based on the T1 data, a confirmatory factor analysis supported the proposed two-factor structure of the scale, *χ*^2^(13) = 38.74, CFI = 0.960, TLI = 0.936, RMSEA = 0.057, and SRMR = 0.035. Standardized factor loadings ranged from 0.589 to 0.665 for positive emotional experiences and from 0.752 to 0.788 for negative emotional experiences. McDonald’s omega coefficients were 0.720 and 0.740, respectively, indicating acceptable reliability and supporting the psychometric adequacy of the measure. Cronbach’s α coefficients for the positive emotional experiences dimension were 0.719 (T1), 0.789 (T2), and 0.784 (T3), whereas the corresponding coefficients for the negative emotional experiences dimension were 0.738 (T1), 0.728 (T2), and 0.701 (T3).

### 2.3. Data Analysis

First, common method bias across the three waves of data was assessed using Harman’s single-factor test. The variance explained by the first factor was 29.64%, 33.54%, and 32.20%, all below the critical threshold of 40% (Podsakoff et al., 2003), suggesting that common method variance was unlikely to substantially affect the findings.

Then we conducted longitudinal measurement invariance testing using MPLUS 8.0 to ensure consistent capture of constructs over time. All models were estimated using maximum likelihood estimation with robust standard errors (MLR). All latent growth models and cross-lagged panel models were estimated using observed composite scores derived from the respective scales rather than latent variables. Means, Standard Deviations, and correlation coefficients for the study variables at each time point were calculated in SPSS 22.0 to provide descriptive information. To examine developmental trends, we employed a latent growth model assuming linear change, given that only three waves were available and more complex nonlinear trajectories could not be reliably identified. This model estimated the intercept and slope as latent variables, allowing us to explore their correlations in growth trajectories. Subsequently, a latent growth model of academic emotions with S-P and gender as covariates was introduced to explore whether gender and S-P would affect the development trend of academic emotions.

Finally, the study adopted a cross-lagged panel design to examine longitudinal predictive associations among variables across the three waves. Specifically, this model assessed whether variables at earlier time points predicted outcomes at later stages, thereby providing evidence regarding the temporal ordering and directional associations among the study variables. The conditional latent growth models and cross-lagged panel models served complementary purposes. The conditional growth models examined concurrent associations between S-P and academic emotions across the three waves, whereas the cross-lagged models evaluated prospective associations by testing whether S-P predicted subsequent changes in academic emotions over time.

For each model, standard goodness-of-fit indices were evaluated, including *χ*^2^, CFI, TLI, and RMSEA. Following L. Hu and Bentler (1999), CFI and TLI values of 0.90 or higher and RMSEA values below 0.08 were considered indicative of acceptable model fit.

## 3. Results

### 3.1. Longitudinal Measurement Invariance

A combined measurement model including all indicators of shift-and-persist, positive emotional experiences, and negative emotional experiences across the three waves was tested. Configural, metric, scalar, and strict invariance models were sequentially compared by imposing increasingly restrictive equality constraints. Correlated residuals were allowed across waves for identical indicators to account for item-specific stability over time. As shown in Table 1, the results supported strict longitudinal invariance (∆CFI < 0.01, ∆TLI < 0.01, ∆RMSEA < 0.015, and ΔSRMR < 0.01).

### 3.2. Descriptive Statistics Results

In Table 2, descriptive statistics and correlations between Shift-and-Persist (S-P), negative emotions (NE), and positive emotions (PE) are reported for each of the three assessment waves. Significant autocorrelations were observed for S-P, NE, and PE at all three time points. Across all time points, NE showed inverse associations with both S-P and PE, while PE was directly associated with S-P.

### 3.3. Analysis of Developmental Trends

Table 3 presents the results of the unconditional latent growth models for each variable. For negative emotions (NE), the model showed mixed but overall acceptable fit. Specifically, CFI (0.975), TLI (0.924), and SRMR (0.030) indicated satisfactory model fit, whereas RMSEA (0.100) exceeded the conventional cutoff value. However, the model had only one degree of freedom, and previous methodological research has suggested that RMSEA may overestimate model misfit in models with very small degrees of freedom and therefore should not be interpreted in isolation (Kenny et al., 2015). Considering the overall pattern of fit indices, the model was deemed acceptable for subsequent interpretation. Both the intercept and slope were significant, indicating that negative emotions increased over time. The correlation between the intercept and slope was not statistically significant.

For positive emotions (PE), the model demonstrated excellent fit (CFI = 1.000, TLI = 1.000, RMSEA = 0.000, SRMR = 0.003). The intercept was significant, whereas the slope was nonsignificant, suggesting relative stability in positive emotions across the study period. The correlation between the intercept and slope was also nonsignificant.

### 3.4. Latent Growth Model of Academic Emotions with S-P and Gender as Covariates

**Figure 1:**
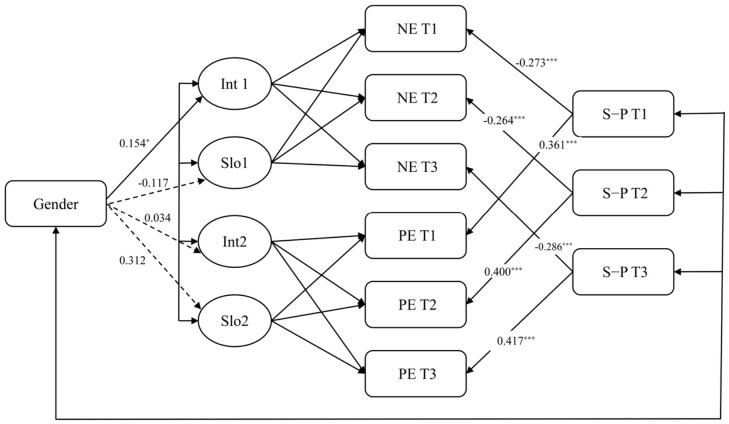
Latent Growth Model of Academic Emotions with S-P and Gender as Covariates. Dashed arrows, non-significant paths; solid arrows, significant paths. * = *p* < 0.05, *** = *p* < 0.001.

To investigate the effects of gender and S-P on the developmental trends of academic emotions among high school students, we specified a conditional growth model to examine these effects (as illustrated in Figure 1), with S-P serving as a time-varying covariate and gender as a time-invariant covariate. The results indicated that the model fit indices of this model were acceptable (*χ*^2^ = 40.073; *df* = 21; CFI = 0.981; TLI = 0.965; SRMR = 0.045; RMSEA = 0.039).

After incorporating gender and S-P as covariates into the latent growth model, the intercepts of both negative emotions (*I* = 8.42, *p* < 0.001) and positive emotions (*I* = 7.44, *p* < 0.001) remained significant, whereas the slopes were no longer statistically significant (negative emotions: *S* = 1.59, *p* = 0.103; positive emotions: *S* = −3.64, *p* = 0.070). Notably, although the unconditional growth model indicated a significant increase in negative academic emotions over time, this effect was attenuated and became non-significant after the inclusion of gender and S-P. This change suggests that part of the variance previously captured by the growth slope was accounted for after controlling for these covariates. Therefore, the difference between the unconditional and conditional models should not be interpreted as contradictory; rather, it indicates that the estimated growth trajectory was influenced by individual difference variables included in the conditional model.

When gender was included as a covariate, the analysis revealed that gender significantly predicted the intercept of negative emotions. Specifically, girls exhibited a higher initial level of negative emotions (*M* = 2.23, *SD* = 0.52) compared to boys (*M* = 2.08, *SD* = 0.57). However, gender did not significantly influence the slope of negative emotions or the intercept and slope of positive emotions.

At the same measurement points, results from the conditional growth model indicated that shift-and-persist significantly predicted students’ academic emotions, being associated with lower negative emotions and higher positive emotions. This pattern indicates that S-P plays an important role in shaping students’ emotional experiences, such that higher levels of S-P correspond to more favorable emotional outcomes. Building on this, a cross-lagged panel model was then conducted to examine whether S-P prospectively predicted emotional outcomes over time and to test the possibility of reciprocal associations.

### 3.5. Longitudinal Association Between S-P, NE and PE

Cross-lagged panel analysis controls for the autoregressive effects of each variable by incorporating stability coefficients, allowing researchers to examine directional prospective associations between variables over time (Preacher, 2015). Therefore, to examine directional longitudinal associations between variables, we tested four competing models: (1) M1, the baseline model, includes only autoregression to assess the stability of the variables at each time point; (2) M2 builds on M1 by adding paths from S-P at the previous time point to PE and NE at the subsequent time point; (3) M3, also based on M1, adds paths from PE and NE at the previous time point to S-P at the next time point; and (4) M4 is the full model that incorporates all paths from the preceding models.

Table 4 summarizes the fit statistics and chi-square difference tests for the four competing models. Compared to the autoregressive model (M1), adding cross-lagged paths significantly improved fit for M2 (Δ*χ*^2^(5) = 68.15, *p* < 0.001) and M4 (Δ*χ*^2^(8) = 42.19, *p* < 0.001), but not for M3 (Δ*χ*^2^(4) = 5.13, *p* = 0.274). These findings suggest that including prospective paths from shift-and-persist to subsequent academic emotions improved the representation of the longitudinal associations relative to the autoregressive model.

Although the chi-square difference test between M2 and M4 was statistically significant (Δ*χ*^2^(3) = 25.96, *p* < 0.001), the additional reciprocal paths specified in M4 (from academic emotions to subsequent shift-and-persist) were all non-significant (T1 PE → T2 S-P: *β* = 0.03, *p* = 0.55; T1 NE → T2 S-P: *β* = −0.04, *p* = 0.20; T2 PE → T3 S-P: *β* = 0.03, *p* = 0.49; T2 NE → T3 S-P: *β* = 0.01, *p* = 0.71). Given that these paths did not add substantive explanatory value, M2 was retained as the preferred model based on the principle of parsimony. However, as the overall fit of M2 was acceptable rather than excellent (CFI = 0.940, TLI = 0.876, RMSEA = 0.086), the results derived from this model should be interpreted with appropriate caution.

**Figure 2:**
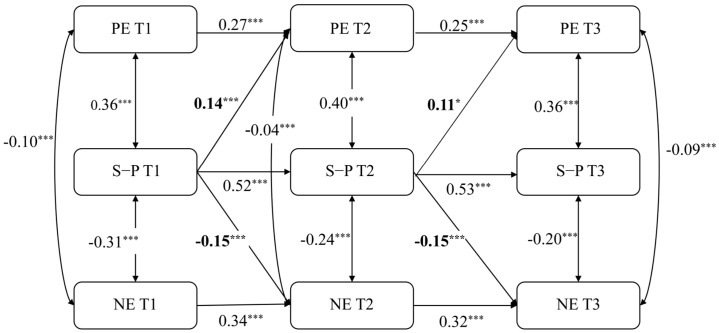
Significant paths. Numbers are Standardized Coefficients. * = *p* < 0.05, *** = *p* < 0.001.

As illustrated in Figure 2, the autoregressive paths show that the path coefficients between each variable at all time points are significant, indicating that S-P strategies, along with PE and NE, exhibit stability over time. Importantly, S-P significantly and negatively predicted NE one year later (T2: *β* = −0.152, *SE* = 0.038, *p* < 0.001, 95% CI [−0.23, −0.08]; T3: *β* = −0.149, *SE* = 0.039, 95% CI [−0.23, −0.08], *p* < 0.01). In contrast, S-P significantly and positively predicted PE one year later (T2: *β* = 0.140, *SE* = 0.040, 95% CI [0.06, 0.22], *p* < 0.001; T3: *β* = 0.105, *SE* = 0.041, 95% CI [0.03, 0.19], *p* < 0.001).

## 4. Discussion

This study adopted a longitudinal design, applying latent growth and cross-lagged panel models to explore the developmental course of academic emotions in high school students and their prospective links with shift-and-persist strategies. Findings indicated that negative academic emotions increased steadily across years, whereas positive emotions remained relatively stable. Moreover, at the concurrent level, greater use of shift-and-persist was associated with more favorable emotional profiles, including elevated positive emotions and reduced negative emotions. Cross-lagged results indicated that higher levels of S-P were associated with more favorable emotional outcomes at subsequent time points.—promoting higher positive emotions and attenuating negative ones—while academic emotions themselves did not show evidence of influencing subsequent S-P use. These findings are discussed in detail below.

### 4.1. Developmental Trajectories of Academic Emotions in High School Students

The findings partially supported Hypothesis 1. Specifically, negative academic emotions displayed a significant upward trajectory, consistent with prior evidence that upper-grade students report more frequent negative feelings in school, while positive academic emotions declined slightly but without a clear downward trend. Similar trajectories have been identified in prior research (Jin et al., 2022; Raccanello et al., 2013; Wang et al., 2015). The escalation of academic workload and evaluative pressure across grades may serve as a key reason contributing to the rise in negative emotions. As students approach the highly competitive National College Entrance Examination (Gaokao), they face greater academic demands, increased course difficulty, extended study hours, and frequent tests and evaluations. These stressors can place students in a sustained state of high pressure, leading to heightened anxiety, frustration, and other negative emotions (Pekrun et al., 2006).

Moreover, high parental expectations—common in Chinese families—may translate into psychological pressure, particularly when students fail to meet academic goals. This can further intensify feelings of guilt and anxiety (Wilkinson-Lee et al., 2011). In addition, adolescents are still developing emotional regulation capacities (Casey et al., 2010), and this developmental stage is associated with increased vulnerability to internalizing problems such as anxiety, depression, and self-injury (Beesdo et al., 2010; Young et al., 2019).

Importantly, the observed increase in negative emotions does not necessarily imply a corresponding decrease in positive emotions. Rather, positive emotions remained relatively stable over time, suggesting that students may still access or cultivate positive emotional experiences—potentially through adaptive coping strategies such as shift-and-persist. These longitudinal findings extend prior work by offering a more nuanced understanding of the developmental trajectories of academic emotions throughout the high school years.

### 4.2. Roles of Gender and Shift-and-Persist in Academic Emotion Trajectories

A gender difference was observed in the initial levels of negative academic emotions, with girls exhibiting higher levels than boys. However, the rate of change over time did not differ by gender. The significant gender effect on the intercept suggests that girls begin high school with higher levels of negative emotions than boys (Huang et al., 2026). This finding is consistent with prior evidence that female students may be more vulnerable to academic stress and examination-related anxiety (Goetz et al., 2013; Jacques & Mash, 2004; Pekrun, 2017). These patterns may be interpreted in terms of gender differences in emotional expression and sociocultural norms (Fischer et al., 2004), whereby girls may be more likely to report negative emotions, whereas boys may suppress or underreport them due to norms discouraging emotional expression. However, as these mechanisms were not directly examined, this interpretation remains tentative. Alternatively, the gender difference may reflect measurement-related factors, such as differences in self-report tendencies or social desirability bias rather than true emotional differences. Gender influenced only the initial level of negative academic emotions, not their developmental trajectories. Notably, the increase in negative emotions observed in the unconditional model became non-significant after including S-P and gender as covariates, suggesting that developmental changes may be partly explained by individual differences.

Additionally, higher levels of shift-and-persist were concurrently associated with more favorable emotional profiles, characterized by higher positive emotions and lower negative emotions. These findings are consistent with the view that shift-and-persist represents an adaptive psychological coping strategy associated with lower negative emotions and higher positive emotions (Benner et al., 2024; Lopez-Cepero et al., 2024). By promoting cognitive reappraisal and maintaining optimism, shift-and-persist strategies help students reinterpret stressors in a more constructive way, thereby fostering more positive academic experiences (Brockman et al., 2017). When facing exam-related stress, students who employ shift-and-persist are more likely to view pressure as an opportunity for growth rather than as a threat. Moreover, this strategy enhances psychological resilience and self-efficacy by encouraging an optimistic outlook, which helps students maintain a positive mindset in the face of academic challenges (E. Chen & Miller, 2012).

These results are consistent with the control-value theory of academic emotions, which posits that internal coping resources can influence emotional experiences by shaping individuals’ cognitive appraisals of academic situations (Pekrun et al., 2006). From a complementary motivational perspective, Self-Determination Theory (Ryan & Deci, 2020) further suggests that coping strategies such as shift-and-persist may support students’ sense of agency and competence when facing academic stress, thereby facilitating more adaptive emotional adjustment. Our findings highlight shift-and-persist as a potentially important psychological resource associated with emotional adjustment among high school students experiencing academic stress.

### 4.3. Cross-Lagged Associations Between Shift-and-Persist and Academic Emotions

Shift-and-persist strategies were prospectively associated with higher positive academic emotions and lower negative academic emotions over time, although the effect sizes were relatively modest. In contrast, academic emotions did not significantly predict later use of shift-and-persist strategies. These findings extend the control-value theory of academic emotions by emphasizing the role of internal cognitive and emotion regulation strategies in shaping students’ emotional experiences (Pekrun et al., 2007). They are also consistent with prior research reporting beneficial associations between shift-and-persist and individuals’ mental and physical well-being (Benner et al., 2024; Lopez-Cepero et al., 2024; Santo et al., 2024).

The absence of a significant cross-lagged effect from academic emotions to shift-and-persist may reflect the developmental origins of this coping strategy. Prior studies suggest that shift-and-persist begins to form in childhood, primarily through parental modeling (E. Chen & Miller, 2012; Christophe et al., 2024). Parents contribute to the development of adaptive emotion regulation by modeling appropriate social behaviors, fostering secure attachment relationships, and explicitly teaching coping strategies (Lee et al., 2024; Mello et al., 2020). As a result, academic emotions experienced during high school may not be strong drivers of change in the use of shift-and-persist strategies.

These findings suggest that shift-and-persist may represent a psychological resource associated with more favorable emotional outcomes over time, although the observed effects were modest. From an applied perspective, these results highlight the potential value of focusing on students’ adaptive coping processes in understanding emotional adjustment during high school. However, given the correlational nature of the present study, any implications for educational practice or intervention development should be interpreted with caution. Future research may further examine whether and how such coping-related strategies can be supported or cultivated within school and family contexts using experimental or intervention designs. By examining academic emotions across the entire three-year high school period and their longitudinal associations with shift-and-persist, this study contributes to a more comprehensive understanding of emotional adaptation in high-pressure educational contexts.

### 4.4. Limitations and Recommendations for Future Research

This research represents the first attempt to combine latent growth modeling with cross-lagged panel analysis in a longitudinal framework to investigate the developmental course of academic emotions and the role of shift-and-persist strategies in high school students. Nonetheless, certain constraints should be noted. First, the sample was drawn from a single school, which limits generalizability. Future research should include more diverse samples and consider contextual factors such as academic achievement, socioeconomic status, parental expectations, family support, classroom climate, and school-related stressors that may jointly influence adolescents’ emotions and coping processes. Second, the exclusive reliance on self-report instruments raises concerns about potential response biases, such as the tendency to present oneself in a socially desirable way, which may create discrepancies between reported experiences and actual behavior. Future studies could incorporate experimental or observational methods to better examine causal relationships. Third, although measurement invariance was established, the use of observed-variable models does not fully account for measurement error, and standard cross-lagged panel models cannot fully disentangle between-person differences from within-person processes, nor rule out the influence of unmeasured confounding variables, which limits causal interpretation of the observed associations. Fourth, the variable-centered design and brief scales may limit the depth of assessment and fail to capture heterogeneity in developmental trajectories; person-centered approaches may provide additional insight. Finally, the one-year interval between assessments may not have been sensitive enough to detect finer-grained changes in academic emotions. More frequent or shorter-interval assessments may help capture more nuanced emotional dynamics over time.

## 5. Conclusions

The current study found that negative academic emotions increased over time, while positive emotions remained relatively stable. Notably, shift-and-persist strategies were associated with more adaptive emotional profiles—higher positive emotions and lower negative emotions—at each time point and were prospectively associated with more favorable emotional outcomes over time. These findings suggest that shift-and-persist may represent a beneficial psychological resource associated with adolescents’ emotional adjustment during high school, although these associations were modest in magnitude. By examining academic emotions across the entire three-year high school period and exploring their longitudinal associations with shift-and-persist strategies, the present study extends existing research on academic emotions and contributes to a more comprehensive understanding of emotional adaptation in high-pressure educational contexts.

## Figures and Tables

**Table 1:** Fit Statistics for Measurement Model and Tests of Measurement Invariance.

Model	*χ* ^2^	*df*	CFI	TLI	SRMR	RMSEA
Configural Invariance	105.756	39	0.970	0.950	0.034	0.053
Metric Invariance	113.613	45	0.970	0.955	0.039	0.050
Scalar Invariance	127.943	46	0.964	0.948	0.042	0.054
Strict Invariance	145.412	55	0.960	0.952	0.050	0.052

**Table 2:** Means, Standard Deviations and Correlations of the Assessed Variables.

	*M*	*SD*	1	2	3	4	5	6	7	8	9	10
1. S-P T1	2.73	0.50	—									
2. S-P T2	2.69	0.54	0.52 **	—								
3. S-P T3	2.70	0.52	0.43 **	0.53 **	—							
4. NE T1	2.15	0.55	−0.31 **	−0.24 **	−0.13 **	—						
5. NE T2	2.32	0.62	−0.26 **	−0.35 **	−0.21 **	0.41 **	—					
6. NE T3	2.36	0.61	−0.18 **	−0.26 **	−0.30 **	0.35 **	0.40 **	—				
7. PE T1	2.78	0.47	0.37 **	0.25 **	0.21 **	−0.40 **	−0.29 **	−0.22 **	—			
8. PE T2	2.68	0.54	0.24 **	0.46 **	0.28 **	−0.23 **	−0.28 **	−0.23 **	0.35 **	—		
9. PE T3	2.56	0.56	0.22 **	0.23 **	0.41 **	−0.18 **	−0.21 **	−0.39 **	0.35 **	0.37 **	—	
10. Gender	1.50	0.50	−0.16 **	−0.16 **	−0.08 **	−0.14 *	0.01	0.08 *	0.13 *	0.11 *	0.06	—

*Note*. S-P = Shift-and-Persist, NE = Negative Emotion, PE = Positive Emotion, * = *p* < 0.05, ** = *p* < 0.01. Gender is a dummy variable (1 = male, 2 = female).

**Table 3:** Unconditional Growth Models for NE and PE.

	*χ*^2^/*df*	*p*	CFI	TLI	RMSEA	SRMR	Intercept	Slope	The Correlation Between I and S
NE	7.104	0.07	0.975	0.924	0.100	0.030	5.498 ***	0.665 ***	−0.305
PE	0.102	0.75	1.000	1.000	0.000	0.003	9.611 ***	−1.236	0.166

*Note*. NE = Negative Emotion, PE = Positive Emotion, *** = *p* < 0.001.

**Table 4:** Model Fit Indices.

Model	*χ* ^2^	*df*	CFI	TLI	SRMR	RMSEA	Model Comparison	∆*χ*^2^	∆*df*	*p*
M1	156.580	21	0.890	0.820	0.100	0.103				
**M2**	**88.425**	**16**	**0.940**	**0.876**	**0.062**	**0.086**	**M1 vs. M2**	**68.15**	**5**	**0.000**
M3	151.448	17	0.888	0.783	0.096	0.114	M1 vs. M3	5.13	4	0.274
M4	114.393	13	0.916	0.786	0.062	0.113	M1 vs. M4	42.19	8	0.000

*Note*. Bold indicates the best model.

## Data Availability

The data that support the findings of this study are available from the corresponding author upon reasonable request.
